# Pharmacogenomics, Race, and Treatment Outcome in Pediatric Acute Myeloid Leukemia

**DOI:** 10.1001/jamanetworkopen.2024.11726

**Published:** 2024-05-16

**Authors:** Jatinder K. Lamba, Richard Marrero, Huiyun Wu, Xueyuan Cao, Phani Krishna Parcha, Seth E. Karol, Hiroto Inaba, Dennis John Kuo, Barbara A. Degar, Kenneth Heym, Jeffrey W. Taub, Norman J. Lacayo, Ching-Hon Pui, Raul C. Ribeiro, Stanley B. Pounds, Jeffrey E. Rubnitz

**Affiliations:** 1 Department of Pharmacotherapy and Translational Research, College of Pharmacy, University of Florida, Gainesville; 2 University of Florida Health Cancer Center, University of Florida, Gainesville; 3 Center for Pharmacogenomics and Precision Medicine, College of Pharmacy, University of Florida, Gainesville; 4 Department of Biostatistics, St Jude Children’s Research Hospital, Memphis, Tennessee; 5 Department of Health Promotion and Disease Prevention, University of Tennessee Health Science Center, Memphis; 6 Department of Oncology, St Jude Children’s Research Hospital, Memphis, Tennessee; 7 Division of Pediatric Hematology-Oncology, Rady Children’s Hospital San Diego/University of California, San Diego; 8 Hematology/Oncology, Dana-Farber Cancer Institute, Boston, Massachusetts; 9 Hematology/Oncology, Boston Children’s Hospital, Boston, Massachusetts; 10 Hematology/Oncology, Cook Children’s Medical Center, Fort Worth, Texas; 11 Hematology/Oncology, Children’s Hospital of Michigan, Detroit; 12 Hematology/Oncology, Lucile Packard Children’s Hospital, Palo Alto, California; 13 Hematology/Oncology, Stanford Cancer Institute, Palo Alto, California

## Abstract

**Importance:**

Disparities in outcomes exist between Black and White patients with acute myeloid leukemia (AML), with Black patients experiencing poorer prognosis compared with their White counterparts.

**Objective:**

To assess whether varying intensity of induction therapy to treat pediatric AML is associated with reduced disparities in treatment outcome by race.

**Design, Setting, and Participants:**

A comparative effectiveness analysis was conducted of 86 Black and 359 White patients with newly diagnosed AML who were enrolled in the AML02 trial from 2002 to 2008 or the AML08 trial from 2008 to 2017. Statistical analysis was conducted from July 2023 through January 2024.

**Interventions:**

Patients in AML02 were randomly assigned to receive standard low-dose cytarabine-based induction therapy or augmented high-dose cytarabine-based induction therapy, whereas patients in AML08 received high-dose cytarabine-based therapy.

**Main Outcomes and Measures:**

Cytarabine pharmacogenomic 10–single-nucleotide variant (ACS10) scores were evaluated for association with outcome according to race and treatment arm.

**Results:**

This analysis included 86 Black patients (mean [SD] age, 8.8 [6.5] years; 54 boys [62.8%]; mean [SD] leukocyte count, 52 600 [74 000] cells/µL) and 359 White patients (mean [SD] age, 9.1 [6.2] years; 189 boys [52.6%]; mean [SD] leukocyte count, 54 500 [91 800] cells/µL); 70 individuals with other or unknown racial and ethnic backgrounds were not included. Among all patients without core binding factor AML who received standard induction therapy, Black patients had significantly worse outcomes compared with White patients (5-year event-free survival rate, 25% [95% CI, 9%-67%] compared with 56% [95% CI, 46%-70%]; *P* = .03). By contrast, among all patients who received augmented induction therapy, there were no differences in outcome according to race (5-year event-free survival rate, Black patients, 50% [95% CI, 38%-67%]; White patients, 48% [95% CI, 42%-55%]; *P* = .78). Among patients who received standard induction therapy, those with low ACS10 scores had a significantly worse 5-year event-free survival rate compared with those with high scores (42.4% [95% CI, 25.6%-59.3%] and 70.0% [95% CI, 56.6%-83.1%]; *P* = .004); however, among patients who received augmented induction therapy, there were no differences in 5-year event-free survival rates according to ACS10 score (low score, 60.6% [95% CI, 50.9%-70.2%] and high score, 54.8% [95% CI, 47.1%-62.5%]; *P* = .43).

**Conclusions and Relevance:**

In this comparative effectiveness study of pediatric patients with AML treated in 2 consecutive clinical trials, Black patients had worse outcomes compared with White patients after treatment with standard induction therapy, but this disparity was eliminated by treatment with augmented induction therapy. When accounting for ACS10 scores, no outcome disparities were seen between Black and White patients. Our results suggest that using pharmacogenomics parameters to tailor induction regimens for both Black and White patients may narrow the racial disparity gap in patients with AML.

## Key Points

**Question:**

Can tailoring the intensity of induction therapy mitigate racial disparities in the outcome of pediatric patients with acute myeloid leukemia (AML)?

**Findings:**

In this comparative effectiveness analysis of 86 Black and 359 White patients with AML, Black patients exhibited a higher prevalence of low cytarabine pharmacogenomic 10–single-nucleotide variant (ACS10) scores, which were associated with an unfavorable outcome after initial treatment with low-dose cytarabine-based induction therapy. Augmentation of induction therapy was associated with improved outcome for patients with low ACS10 scores and with no disparity in outcome between Black patients and White patients.

**Meaning:**

This study suggests that discrepancies in pharmacogenomics may explain the differences in outcomes between Black and White patients with AML, providing a strategy to overcome these disparities.

## Teaser

This comparative effectiveness analysis examines the outcomes of pediatric patients with acute myeloid leukemia (AML) by race and cytarabine pharmacogenomics.

## Introduction

Although the outcome of patients with hematologic malignant neoplasms has improved, racial and ethnic disparities, some of which can be attributed to differences in social determinants of health, persist.^1^ Disparities in outcomes among individuals with acute myeloid leukemia (AML) have been observed across age groups, with Black patients experiencing poorer prognosis compared with their White counterparts among children,^2,3,4^ adolescents and young adults,^5^ and adults.^6,7,8^ For instance, Black children treated in the CCG2961 trial from 1996 to 2002 had a significantly lower overall survival rate compared with White children (45% compared with 60%; *P* = .007).^2^ Similar patterns emerged from the Surveillance, Epidemiology, and End Results (SEER) database, where survival rates from 2001 to 2007 stood at 46% for Black children and 67% for White children (*P* < .01).^9^ Furthermore, in a study of 1122 children treated for AML from 2004 to 2014, Black patients had a notably higher mortality rate than White patients during the initial course of therapy (4.9% compared with 1.9%; *P* = .04).^4^ A comprehensive analysis of 814 pediatric patients with AML (108 Black, 154 Hispanic, and 552 non-Hispanic White) treated in cooperative group studies from 1996 to 2013 revealed that Black race was associated with inferior survival in the entire cohort as well as in analyses limited to patients with *KMT2A* rearrangements or core binding factor leukemia (*RUNX1*::*RUNX1T1* or *CBFB*::*MYH11*).^3^

A recent comparison of 89 Black and 566 White adolescent and young adult patients with AML demonstrated that Black patients, especially those 18 to 29 years of age, experienced worse outcomes.^5^ These outcomes included a higher early mortality rate (16% compared with 3%; *P* = .002), lower complete remission rate (66% compared with 83%; *P* = .01), and worse overall survival (22% compared with 51%; *P* < .001), despite a higher prevalence of the favorable *RUNX1*::*RUNX1T1* fusion transcript among Black patients (22% compared with 10%; *P* = .002). These findings emphasize the need to address and rectify the disparities in AML among different racial and ethnic groups.

Racial and ethnic disparities in treatment outcomes are undoubtedly complex, associated with a combination of socioeconomic and biological factors. The increased induction mortality observed among Black patients may be associated with their greater acuity at diagnosis and increased reliance on intensive care support.^4^ Moreover, disparities persist in clinical trial enrollment, with Black patients with AML less likely to enroll in clinical trials compared with White counterparts, a factor potentially associated with the observed discrepancies within the SEER database.^10^ Despite these socioeconomic considerations, the persistent inferior survival of Black patients relative to White patients, even when treated in the same clinical trial, underscores the potential effect of biological differences. To unravel the multifaceted causes of outcome disparities and to develop treatment strategies to overcome these disparities, we conducted a comparative analysis of the outcomes of Black and White pediatric patients with AML treated across 2 consecutive multi-institutional clinical trials. Our focus centered on exploring the associations between race, treatment modalities, and cytarabine pharmacogenomics.

## Methods

This comparative effectiveness study used the existing clinical trial databases of AML02^11^ (NCT00136084), conducted from 2002 to 2008, and AML08^12^ (NCT00703820), conducted from 2008 to 2017, in which pediatric patients with newly diagnosed AML were enrolled. The report adheres to the International Society for Pharmacoeconomics and Outcomes Research (ISPOR) reporting guideline. As previously described, patients enrolled in AML02 were randomly assigned to receive standard induction therapy consisting of low-dose cytarabine, daunorubicin, and etoposide or augmented induction therapy with high-dose cytarabine, daunorubicin, and etoposide. All patients treated in AML08 received augmented induction therapy with high-dose cytarabine, daunorubicin, and etoposide or clofarabine and high-dose cytarabine as initial therapy. Details regarding therapy, risk classification, and outcomes have been previously reported.^11,12^ Given the similarities in supportive care measures, risk classification, and outcomes between AML02 and AML08, the data from both trials were combined for the present analysis. Patients underwent bone marrow aspiration for the assessment of morphologic response and minimal residual disease (MRD) on day 22 of therapy, the results of which were used for risk classification and treatment assignment. Minimal residual disease was determined by flow-cytometric assessment of leukemia-specific immunophenotypes that were identified in diagnostic specimens, with MRD positivity defined as 0.1% or more. The protocols were approved by the institutional review boards of all participating institutions, and written informed consent was obtained from the patients’ guardians or parents, and assent from the patients, in accordance with the principles of the Declaration of Helsinki.^13^

### Reporting Race and Ethnicity

Information on race and ethnicity, as defined by patients or their legal guardians, was collected at the time of enrollment in the AML02 and AML08 clinical trials. Race and ethnicity categories were African American or Black, American Indian or Alaska Native, Asian, Native Hawaiian or Other Pacific Islander, White, and not specified. This information was collected to establish the baseline demographic characteristics of the patients in the AML02 and AML08 studies to examine any associations with outcome.

### Genotyping

For pharmacogenomics studies, genotype information and the polygenic cytarabine pharmacogenomic 10–single-nucleotide variant (ACS10) score for patients in the AML02 trial were derived from a combination of 10 single-nucleotide variants (SNVs) and were computed as previously reported.^14^ For the AML08 trial cohort, genomic DNA was genotyped using the Illumina Omni 2.5M Exome Beadchip (Illumina Inc) at Hussman Institute for Human Genetics, University of Miami, Miami, Florida. Six of the 10 SNVs that are part of the ACS10 score were typed on the Illumina 2.5 Omni array. Two (rs17103168 and rs11030918) of the remaining 4 SNVs were not represented in the array and were thus genotyped using TaqMan allele discrimination assay using QuantStudio 5 real-time polymerase chain reaction system (Thermo Fisher Scientific Inc). For the other 2 SNVs, genotype data for SNVs (rs1890005 in linkage disequilibrium with rs10916819 and rs10805074 in linkage disequilibrium with rs4643786) that occurred in high linkage disequilibrium (*D*′ = 1.0 and *r*^2^ = 1.0) were used to calculate the ACS10 score.

### Statistical Analysis

Statistical analysis was conducted from July 2023 through January 2024. Patients’ characteristics were computed as mean (SD) values for continuous variables and as frequency and percentage for categorical variables by race. Event-free survival was defined as the time from study entry to induction failure, relapse, secondary malignant neoplasm, or death, with event-free patients censored on the date of last follow-up. Overall survival was defined as the time from study enrollment to death, with living patients censored on the date of last follow-up. Event-free and overall survival probabilities were estimated using the Kaplan-Meier method.^15^ Cox proportional hazards regression models^16^ were used to associate ACS10 score with event-free and overall survival. Multivariable Cox proportional hazards regressions were also used to model event-free and overall survival with ACS10 groups, risk group assignment, MRD, race, leukocyte count at diagnosis, and age as factors potentially associated with outcomes. The 95% CI of hazard ratios was calculated to quantitatively measure the association with clinical outcome. The Kruskal-Wallis test and the Wilcoxon rank sum test were used to compare the median values of continuous variables across groups. The χ^2^ test and the Fisher exact test were used to evaluate the association among pairs of categorical variables. All *P* values were from 2-sided tests, and results were deemed statistically significant at *P* < .05. All statistical analyses were performed in R Statistical Software, version 4.1.0 (R Project for Statistical Computing).

## Results

Of 515 patients treated in AML02 or AML08, 86 (16.7%) were Black (mean [SD] age, 8.8 [6.5] years; 54 boys [62.8%]; mean [SD] leukocyte count, 52 600 [74 000] cells/µL), and 359 (69.7%) were White (mean [SD] age, 9.1 [6.2] years; 189 boys [52.6%]; mean [SD] leukocyte count, 54 500 [91 800] cells/µL); 70 patients (13.6%) with other (American Indian or Alaska Native, Asian, and Native Hawaiian or Other Pacific Islander) or unknown racial and ethnic backgrounds were not included in this analysis (eTable 1 in Supplement 1). There were no statistically significant differences between Black and White patients in age, sex distribution, or leukocyte count at the time of enrollment, but a higher prevalence of core binding factor leukemia was observed among Black patients than White patients (27 of 86 [31.4%] and 72 of 359 [20.1%], respectively; *P* = .04) (eTable 1 in Supplement 1).

**Figure 1.**
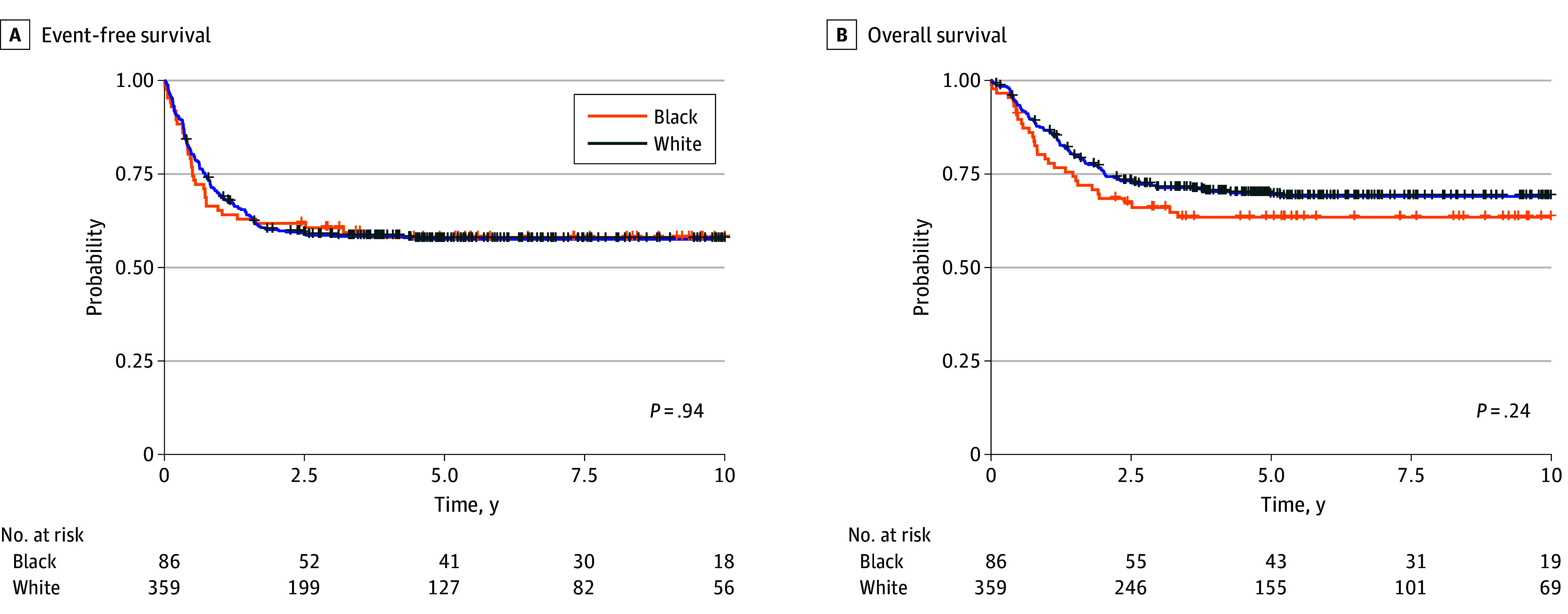
Outcome According to Race

Complete remission rates after 2 courses of induction therapy were comparable between Black and White patients (92.6% [76 of 82] and 95.0% [321 of 338], respectively; *P* = .63), as were the rates of MRD negativity after 1 course of therapy (55.8% [48 of 86] and 55.4% [199 of 359], respectively; *P* = .85). The event-free survival distributions of Black and White patients were similar, with 5-year estimates of 58.3% (95% CI, 48.8%-69.8%) and 58.2% (95% CI, 53.2%-63.6%; *P* = .94), respectively (Figure 1A). Likewise, overall survival did not differ significantly between Black and White patients, with 5-year estimates of 63.8% (95% CI, 54.3%-74.8%) and 69.4% (95% CI, 64.7%-74.5%; *P* = .24), respectively (Figure 1B). In addition, the estimated 5-year cumulative incidence of relapse did not differ by race (26.0% among Black patients and 26.1% among White patients; *P* = .99), nor did the incidence of treatment-related mortality (7.0% among Black patients and 6.8% among White patients; *P* = .78).

Given the higher prevalence of core binding factor leukemia among Black patients compared with White patients and its association with a favorable outcome, we conducted a subgroup analysis by examining outcomes based on race among patients with or without core binding factor leukemia (eFigure 1 in Supplement 1). There were no significant differences in event-free survival rates between Black and White patients with core binding factor leukemia (84.6% [95% CI, 67.1%-100%] and 88.6% [95% CI, 78.6%-99.7%], respectively; *P* = .70) or between Black and White patients without core binding factor leukemia (53.7% [95% CI, 43.4%-66.5%] and 54.9% [95% CI, 49.6%-60.7%], respectively; *P* = .77).

**Figure 2.**
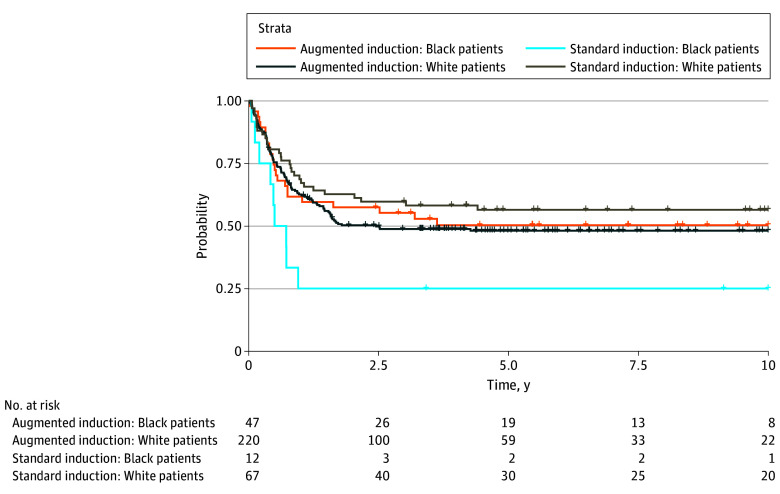
Event-Free Survival According to Race and Treatment for Patients Without Core Binding Factor Leukemia

We next evaluated outcomes according to race and initial induction treatment (standard, low-dose cytarabine-based therapy vs augmented therapy). Patients with core binding factor leukemia had excellent outcomes independent of race or treatment regimen. However, among patients without core binding factor AML who received standard induction therapy, Black patients had significantly worse outcomes compared with White patients (5-year event-free survival rate, 25% [95% CI, 9%-67%] compared with 56% [95% CI, 46%-70%]; *P* = .03) (Figure 2). This disparity was not found among those treated with augmented induction therapy, with 5-year event-free survival rates of 50% (95% CI, 38%-67%) for Black patients and 48% (95% CI, 42%-55%) for White patients (*P* = .78).

**Figure 3.**
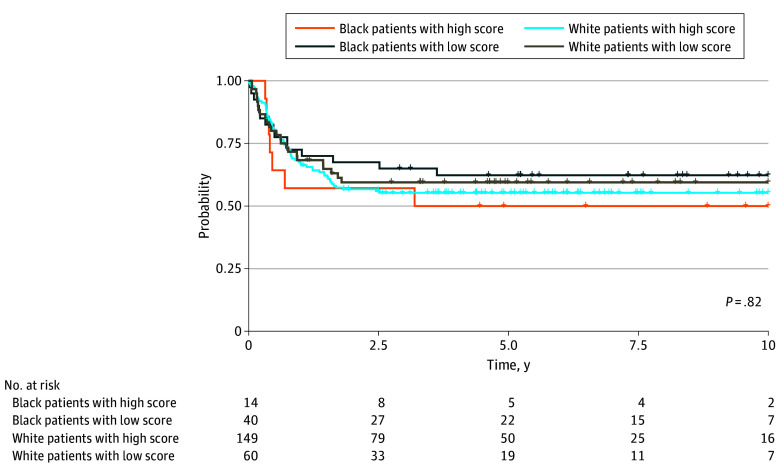
Event-Free Survival According to Race and Cytarabine Pharmacogenomic 10–Single-Nucleotide Variant (ACS10) Score for Patients Treated With Augmented Induction Therapy

To discern the causes of the aforementioned outcome disparities among patients treated with standard therapy, we further evaluated outcomes by race, treatment, and pharmacogenomics, focusing on ACS10, the recently defined cytarabine pharmacogenomics–based polygenic score.^14^ Among the 70 of 86 Black patients (81.4%) and 273 of 359 White patients (76.0%) for whom pharmacogenetic data were available, there was a significant difference in the distribution of ACS10 scores according to race. Specifically, low scores were present for 51 of 70 Black patients (72.9%) compared with 82 of 273 White patients (30.0%) (*P* < .001). When considering patients across all treatment regimens, there were no significant differences in event-free survival distributions between Black and White patients with low ACS10 scores (eFigure 2A in Supplement 1) or between Black and White patients with high ACS10 scores (eFigure 2B in Supplement 1). Among patients who received standard induction therapy, those with low ACS10 scores had significantly worse 5-year event-free survival rates compared with those with high scores (42.4% [95% CI, 25.6%-59.3%] and 70.0% [95% CI, 56.6%-83.1%]; *P* = .004), a finding consistent with a previous study.^14^ By contrast, no significant differences in outcome by ACS10 score were observed in Black or White patients who were treated with augmented induction therapy (low score, 60.6% [95% CI, 50.9%-70.2%] and high score, 54.8% [95% CI, 47.1%-62.5%]; *P* = .43). Neither race nor ACS10 score was associated with outcome among patients who received augmented induction therapy (Figure 3).

## Discussion

Multiple studies have shown that Black patients with AML have worse outcomes compared with non-Hispanic White patients.^2,3,4,5,6,7,8^ For example, in a recent study of adolescent and young adult patients with AML, Black race was independently associated with poor outcome.^5^ This discrepancy was particularly pronounced among patients 18 to 29 years of age without core binding factor leukemia, with 5-year survival rates of 12% for Black patients compared with 44% for White patients (*P* < .001). In light of these reports, we assessed the association of race with patient outcomes within the context of the multisite AML02 and AML08 clinical trials. We combined the trials because they were conducted at largely the same sites, used similar supportive care measures, included similar risk stratification based on genomics and MRD, and produced comparable outcomes. Our pharmacogenomic analyses focused on SNVs in genes involved in cytarabine metabolism because activation of cytarabine to cytarabine-triphosphate and intracellular accumulation of cytarabine-triphosphate are important factors associated with treatment response.^17,18,19,20,21,22^ Low ACS10 scores, which are associated with lower levels of intracellular cytarabine-triphosphate,^19^ were markedly more prevalent among Black patients, accounting for 72.9% of cases, in contrast to just 30.0% among White patients. This divergence was associated predominantly with 3 SNVs exhibiting dissimilar allele frequencies among Black and White patients. Single-nucleotide variant rs4643786 in *DCK*, which is associated with lower intracellular cytarabine-triphosphate levels and worse outcome, was markedly more abundant among Black patients than White patients (minor allele frequency, 0.48 for Black patients and 0.04 for White patients).^14,19^ By contrast, variant alleles rs1044457 in *CMPK1* and rs17343066 in *SLC28A3*, associated with favorable outcomes and higher intracellular cytarabine-triphosphate levels, were notably less common among Black patients compared with White patients (rs1044457, 0.11 among Black patients and 0.5 among White patients; rs17343066, 0.15 among Black patients and 0.53 among White patients).^19^ The distinctive distribution of ACS10 scores according to race takes on particular significance in light of a recent finding that patients with low ACS10 scores had worse outcomes compared with patients with high ACS10 scores when treated with standard, low-dose cytarabine–based induction therapy in the St Jude AML02 trial or the Children’s Oncology Group AAML0531 trial.^14^ In that report, the event-free survival rates for patients with low ACS10 scores were approximately 10 percentage points higher among patients who received augmented induction therapy with high-dose cytarabine in the AML02 trial or with gemtuzumab in the AAML0531 trial compared with those who received standard induction therapy.

Considering the higher prevalence of low ACS10 scores among Black patients and a previous demonstration that the outcomes of patients with low ACS10 scores can be improved by augmenting induction therapy,^14^ we explored potential interactions between race, induction therapy, pharmacogenomics, and outcome in the present study. Overall, we observed no significant differences in outcome between Black and White patients treated in the St Jude AML02 and AML08 trials. In contrast to recent reports,^3,5^ the outcome of Black and White patients with core binding factor was excellent, with no significant differences according to race. However, among patients without core binding factor leukemia who received standard induction therapy, Black patients had significantly worse outcomes compared with White patients. Because both Black patients and White patients with low ACS10 scores had significantly worse outcomes compared with patients with high scores when treated with standard induction therapy, the high prevalence of low ACS10 scores likely explains the racial disparity in outcome for this treatment group. These disparities in outcome were nullified when induction therapy was augmented with high-dose cytarabine or with clofarabine and high-dose cytarabine, after which neither race nor ACS10 score had an association with outcomes. The improved outcome after intensified induction therapy suggests that the poor outcome after standard induction therapy was associated with relative underdosing rather than excessive toxic effects. In addition, the similarity in consolidation therapy regimens among Black patients and White patients further supports the association of early intensification with outcomes.

We propose that the high prevalence of low ACS10 scores among Black patients is also associated with the poor outcomes reported in other studies, which primarily included patients who underwent standard, low-dose cytarabine–based induction therapy in cooperative group trials.^3,5^ This hypothesis is supported by results reported by investigators from the Children’s Oncology Group.^3,14^ Among patients with AML who were treated in the Children’s Cancer Group 2961 trial or the Children’s Oncology Group AAML03P1 or AAML0531 trials, the event-free and overall survival rates for Black patients were only 35% and 52%, respectively, compared with rates of 50% and 71%, respectively, for White patients.^3^ In a multivariable analysis of patients treated in the standard arm of the AAML0531 trial, Black race was identified as independently associated with worse event-free and overall survival.^14^ However, in an analysis limited to patients with low ACS10 scores, treatment in the standard arm, but not race, was associated with inferior event-free survival. In addition, a multivariable analysis of patients treated in the augmented (gemtuzumab) arm of the AAML0531 trial indicated that Black race was no longer associated with a worse outcome.^14^ An analysis of patients with core binding factor leukemia also showed poorer prognosis for Black patients when low-dose cytarabine-based induction was administered, with event-free survival rates for Black patients of 44% and for White patients of 64%.^3^ In this subgroup analysis, gemtuzumab again had a differential association with outcomes among Black patients and completely eliminated the racial disparity in outcome, resulting in event-free survival rates of 69% for both Black and White patients.^3^ A recent analysis of patients treated in the Children’s Oncology Group AAML1031 trial further suggests that augmentation of induction therapy with bortezomib may have similar associations, with worse outcomes for Black patients who were treated on the standard arm but no disparities among patients in the augmented (bortezomib) arm.^23^ We postulate that, in the present study, the overall lack of significant disparities in outcome according to race or ACS10 score is likely associated with administration of augmented therapy for 78% of patients.

### Limitations

This study has some limitations, including the availability of pharmacogenomic data for 343 patients (77.1%) and the small numbers of patients in certain subgroups, such as Black patients with high ACS10 scores who received standard induction therapy and Black patients with genetic alterations other than core binding factor leukemia, thereby hampering our ability to perform additional multivariable analyses. In addition, we did not directly measure intracellular cytarabine triphosphate levels and were thus unable to explore associations between intracellular cytarabine levels, ACS10 scores, race, and outcome. Moreover, although other studies have demonstrated similar results, we do not have a formal validation cohort to directly test our hypothesis. Given that current clinical trials for pediatric AML do not incorporate high-dose cytarabine or clofarabine during induction therapy, it is not feasible to conduct a validation study.

## Conclusions

In this comparative effectiveness study of pediatric patients with AML, Black patients without core binding factor leukemia had significantly worse outcomes compared with White patients after treatment with standard induction therapy. However, among patients treated with augmented therapy, we observed no racial disparities. The associations between race, pharmacogenomics, treatment intensity, and outcome suggest that racial disparities in outcome are associated with variations in pharmacogenomics. We propose that future studies should include the nonrandomized tailoring of induction regimens to pharmacogenomic parameters to improve the outcome of Black and White patients and serve as a pivotal bridge in addressing the racial gap in AML treatment outcomes.

## Notes

Supplement 1. **eTable.** Patient Characteristics by Race **eFigure 1.** Event-Free Survival According to Race for Patients With (Top Curves) and Without (Lower Curves) Core Binding Factor Leukemia **eFigure 2.** Event-Free Survival According to Race and ACS10 Score

Supplement 2.
Data Sharing Statement

## References

[1] Miranda-Galvis M, Tjioe KC, Balas EA, Agrawal G, Cortes JE. Disparities in survival of hematologic malignancies in the context of social determinants of health: a systematic review. Blood Adv. 2023;7(21):6466-6491. doi:10.1182/bloodadvances.2023010690 37639318 PMC10632659

[2] Aplenc R, Alonzo TA, Gerbing RB, ; Children’s Oncology Group. Ethnicity and survival in childhood acute myeloid leukemia: a report from the Children’s Oncology Group. Blood. 2006;108(1):74-80. doi:10.1182/blood-2005-10-4004 16537811 PMC1895824

[3] Conneely SE, McAtee CL, Gupta R, Lubega J, Scheurer ME, Rau RE. Association of race and ethnicity with clinical phenotype, genetics, and survival in pediatric acute myeloid leukemia. Blood Adv. 2021;5(23):4992-5001. doi:10.1182/bloodadvances.2021004735 34619758 PMC9153027

[4] Winestone LE, Getz KD, Miller TP, . The role of acuity of illness at presentation in early mortality in Black children with acute myeloid leukemia. Am J Hematol. 2017;92(2):141-148. doi:10.1002/ajh.24605 27862214 PMC5733783

[5] Larkin KT, Nicolet D, Kelly BJ, . High early death rates, treatment resistance, and short survival of Black adolescents and young adults with AML. Blood Adv. 2022;6(19):5570-5581. doi:10.1182/bloodadvances.2022007544 35788257 PMC9577622

[6] Bhatnagar B, Kohlschmidt J, Mrózek K, . Poor survival and differential impact of genetic features of Black patients with acute myeloid leukemia. Cancer Discov. 2021;11(3):626-637. doi:10.1158/2159-8290.CD-20-1579 33277314 PMC7933110

[7] Abraham IE, Patel AA, Wang H, . Impact of race on outcomes in intermediate-risk acute myeloid leukemia. Cancer Causes Control. 2021;32(7):705-712. doi:10.1007/s10552-021-01422-4 33837498

[8] Abraham IE, Rauscher GH, Patel AA, . Structural racism is a mediator of disparities in acute myeloid leukemia outcomes. Blood. 2022;139(14):2212-2226. doi:10.1182/blood.2021012830 35061876 PMC9710198

[9] Pui CH, Pei D, Pappo AS, . Treatment outcomes in Black and White children with cancer: results from the SEER database and St Jude Children’s Research Hospital, 1992 through 2007. J Clin Oncol. 2012;30(16):2005-2012. doi:10.1200/JCO.2011.40.8617 22547602 PMC3383176

[10] Winestone LE, Getz KD, Rao P, . Disparities in pediatric acute myeloid leukemia (AML) clinical trial enrollment. Leuk Lymphoma. 2019;60(9):2190-2198. doi:10.1080/10428194.2019.1574002 30732497 PMC6685754

[11] Rubnitz JE, Inaba H, Dahl G, . Minimal residual disease–directed therapy for childhood acute myeloid leukaemia: results of the AML02 multicentre trial. Lancet Oncol. 2010;11(6):543-552. doi:10.1016/S1470-2045(10)70090-5 20451454 PMC3171799

[12] Rubnitz JE, Lacayo NJ, Inaba H, . Clofarabine can replace anthracyclines and etoposide in remission induction therapy for childhood acute myeloid leukemia: the AML08 multicenter, randomized phase III trial. J Clin Oncol. 2019;37(23):2072-2081. doi:10.1200/JCO.19.00327 31246522 PMC7001777

[13] World Medical Association. World Medical Association Declaration of Helsinki: ethical principles for medical research involving human subjects. JAMA. 2013;310(20):2191-2194. doi:10.1001/jama.2013.281053 24141714

[14] Elsayed AH, Cao X, Mitra AK, . Polygenic ara-C response score identifies pediatric patients with acute myeloid leukemia in need of chemotherapy augmentation. J Clin Oncol. 2022;40(7):772-783. doi:10.1200/JCO.21.01422 34990262 PMC8887949

[15] Kaplan EL, Meier P. Nonparametric estimation from incomplete observations. J Am Stat Assoc. 1958;53(282):457-481. doi:10.1080/01621459.1958.10501452

[16] Cox DR. Regression models and life-tables. *J R Stat Soc Series B Stat Methodol*. 1972;34(2):187-220. Accessed March 26, 2024. https://www.jstor.org/stable/2985181

[17] Lamba JK, Crews K, Pounds S, . Pharmacogenetics of deoxycytidine kinase: identification and characterization of novel genetic variants. J Pharmacol Exp Ther. 2007;323(3):935-945. doi:10.1124/jpet.107.128595 17855478

[18] Lamba JK. Genetic factors influencing cytarabine therapy. Pharmacogenomics. 2009;10(10):1657-1674. doi:10.2217/pgs.09.118 19842938 PMC2828057

[19] Elsayed AH, Cao X, Crews KR, . Comprehensive ara-C SNP score predicts leukemic cell intracellular ara-CTP levels in pediatric acute myeloid leukemia patients. Pharmacogenomics. 2018;19(14):1101-1110. doi:10.2217/pgs-2018-0086 30088438 PMC6219441

[20] Mitra AK, Crews KR, Pounds S, . Genetic variants in cytosolic 5′-nucleotidase II are associated with its expression and cytarabine sensitivity in HapMap cell lines and in patients with acute myeloid leukemia. J Pharmacol Exp Ther. 2011;339(1):9-23. doi:10.1124/jpet.111.182873 21712425 PMC3186292

[21] Amaki J, Onizuka M, Ohmachi K, . Single nucleotide polymorphisms of cytarabine metabolic genes influence clinical outcome in acute myeloid leukemia patients receiving high-dose cytarabine therapy. Int J Hematol. 2015;101(6):543-553. doi:10.1007/s12185-015-1766-4 25735499

[22] Hyo Kim L, Sub Cheong H, Koh Y, . Cytidine deaminase polymorphisms and worse treatment response in normal karyotype AML. J Hum Genet. 2015;60(12):749-754. doi:10.1038/jhg.2015.105 26354033

[23] Marrero RJ, Shastri VM, Aplenc R, et al. 4314 ACS10—Cytarabine pharmacogenomics score impacts survival in pediatric AML patients treated on AAML1031 trial and associates with outcome differences in black AML patients [abstract 187515]. Presented at: 2023 American Society of Hematology Annual Meeting & Exposition; December 11, 2013; San Diego, California.

