# Error in Byline

**DOI:** 10.1001/jamanetworkopen.2025.0502

**Published:** 2025-02-06

In the Original Investigation titled “Pharmacogenomics, Race, and Treatment Outcome in Pediatric Acute Myeloid Leukemia,”^1^ published May 16, 2024, the fifth author’s middle name was misspelled in the byline; his full name should read Phani Krishna Parcha. This article has been corrected.^1^
