# Supplementary material for: Pharmacogenomics, Race, and Treatment Outcome in Pediatric Acute Myeloid Leukemia
Source: JAMA Netw Open. 2024 May 16;7(5):e2411726. doi: 10.1001/jamanetworkopen.2024.11726 (PMC11099689; doi:10.1001/jamanetworkopen.2024.11726)
Supplement: Supplement 1. — eTable. Patient Characteristics by Race eFigure 1. Event-Free Survival According to Race for Patients With (Top Curves) and Without (Lower Curves) Core Binding Factor Leukemia eFigure 2. Event-Free Survival According to Race and ACS10 Score [file jamanetwopen-e2411726-s001.pdf]

Supplementary Online Content

Lamba JK, Marrero R, Wu H, et al. Pharmacogenomics, race, and treatment outcome in pediatric acute myeloid leukemia. *JAMA Netw Open.* 2024;7(5):e2411726. doi:10.1001/jamanetworkopen.2024.11726

eTable. Patient Characteristics by Race

eFigure 1. Event-Free Survival According to Race for Patients With (Top Curves) and Without (Lower Curves) Core Binding Factor Leukemia

eFigure 2. Event-Free Survival According to Race and ACS10 Score

This supplementary material has been provided by the authors to give readers additional information about their work.

© 2024 Lamba JK et al. *JAMA Network Open.*

eTable 1. Patient Characteristics by Race

	Total (N=515)	White (N=359)	Black (N=86)	Other* (N=70)	Black vs. White p value
**Age**					
Mean (SD)	9.07 (6.23)	9.13 (6.17)	8.76 (6.45)	9.12 (6.33)	0.63
**Gender**					
Female	229 (44.5%)	171 (47.5%)	33 (37.9%)	25 (36.8%)	0.12
Male	286 (55.5%)	189 (52.5%)	54 (62.1%)	43 (63.2%)	
**Initial Risk**					
Low	126 (24.5%)	84 (23.3%)	27 (31.0%)	15 (22.1%)	0.16
High	192 (37.3%)	135 (37.5%)	32 (36.8%)	25 (36.8%)	1
Standard	197 (38.3%)	141 (39.2%)	28 (32.2%)	28 (41.2%)	0.26
**Cytogenetics**					
11q23 rearrangements	130 (25.2%)	96 (26.7%)	17 (19.5%)	17 (25.0%)	0.21
Normal	104 (20.2%)	77 (21.4%)	16 (18.4%)	11 (16.2%)	0.66
Other	172 (33.4%)	117 (32.5%)	28 (32.2%)	27 (39.7%)	1
t(8;21)	60 (11.7%)	35 (9.7%)	13 (14.9%)	12 (17.6%)	0.17
Inv(16)	49 (9.5%)	35 (9.7%)	13 (14.9%)	1 (1.5%)	0.17
**CBF vs non CBF**					
CBF	109 (21%)	72 (19.4%)	27 (29.9%)	13 (19.1%)	**0.04**
Other	406 (79%)	287 (80.6%)	59 (70.1%)	55 (80.9%)	
**Leukocyte count**					
Mean (SD)	54.2 (89.5)	54.4 (91.8)	52.6 (74.0)	55.0 (96.8)	0.23
**Treatment arm**					
HD-ADE	269 (52.2%)	195 (54.2%)	43 (49.4%)	31 (45.6%)	0.48
Clo/Ara-C	129 (25.0%)	81 (22.5%)	22 (25.3%)	26 (38.2%)	0.57
LD-ADE	117 (22.7%)	84 (23.3%)	22 (25.3%)	11 (16.2%)	0.68
**Clinical Trial**					
AML02	230 (44.7%)	160 (44.7%)	43 (50.6%)	27 (36.8%)	0.34
AML08	285 (55.3%)	199 (55.3%)	43 (49.4%)	43 (63.2%)	

*Other includes American Indian or Alaska Native, Asian, Native Hawaiian or Other Pacific Islander, or unknown
SD, standard deviation; CBF, core binding factor

© 2024 Lamba JK et al. *JAMA Network Open.*

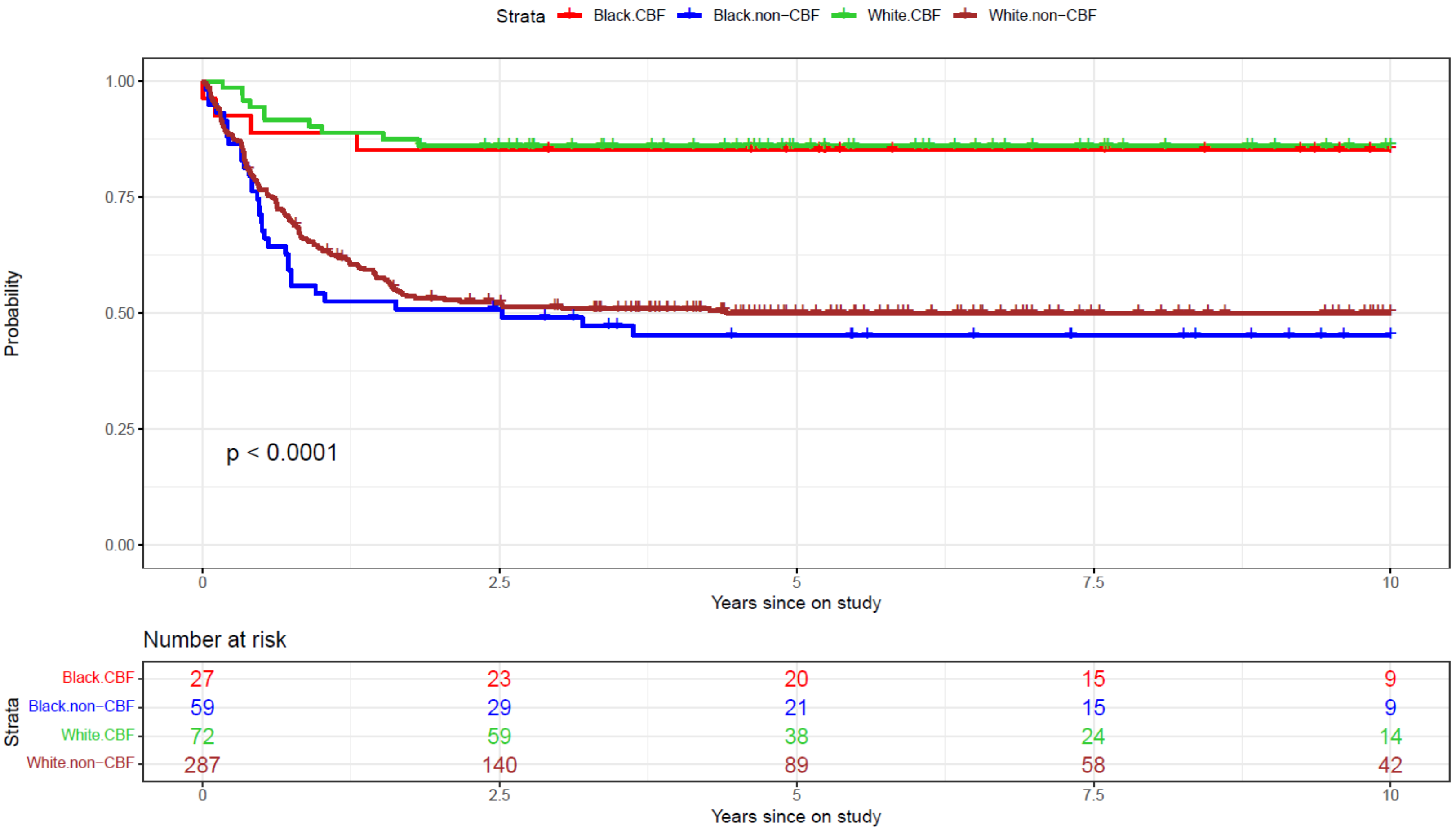

eFigure 1. Event-Free Survival According to Race for Patients With (Top Curves) and Without (Lower Curves) Core Binding Factor Leukemia

© 2024 Lamba JK et al. *JAMA Network Open.*

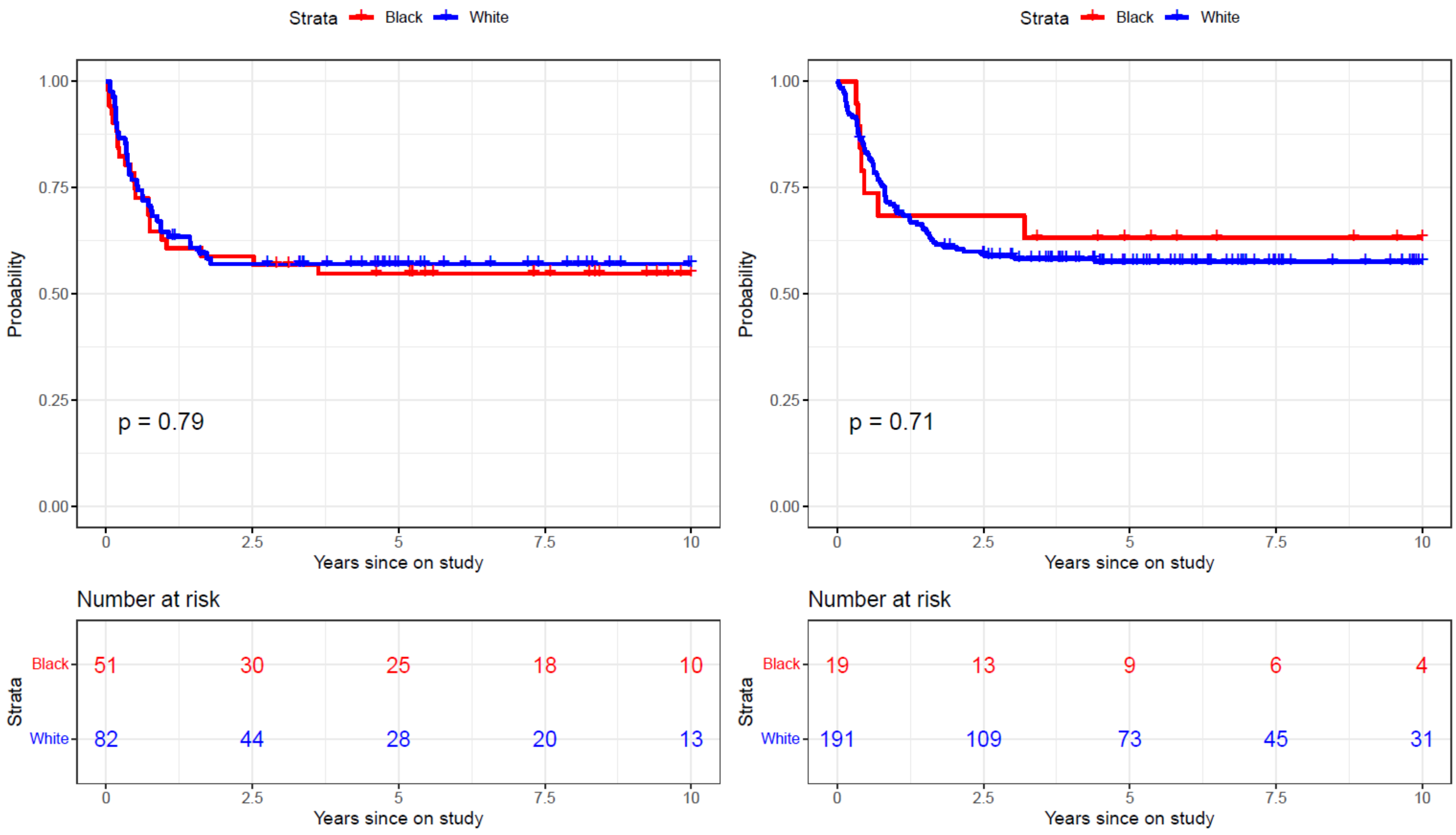

eFigure 2. Event-Free Survival According to Race and ACS10 Score. Left panel: Event-free survival in patients with low ACS10 scores. Right panel: Event-free survival in patients with high ACS10 scores.

© 2024 Lamba JK et al. *JAMA Network Open.*
