# Supplementary material for: Pharmacogenomics, Race, and Treatment Outcome in Pediatric Acute Myeloid Leukemia
Source: JAMA Netw Open. 2024 May 16;7(5):e2411726. doi: 10.1001/jamanetworkopen.2024.11726 (PMC11099689; doi:10.1001/jamanetworkopen.2024.11726)
Supplement: Supplement 2. — Data Sharing Statement [file jamanetwopen-e2411726-s002.pdf]

Data Sharing Statement

Lamba. Pharmacogenomics, Race, and Treatment Outcome in Pediatric Acute Myeloid Leukemia. *JAMA Netw Open*. Published May 16, 2024.
doi:10.1001/jamanetworkopen.2024.11726

Data
Data available: Yes
Data types: Deidentified participant data
How to access data: Deidentified individual participant data will be made available at the time of publication for a period of 5 years after the publication date. Proposals for access should be sent to Jeffrey.rubnitz@stjude.org.
When available: With publication

Supporting Documents
Document types: None

Additional Information
Who can access the data: Anyone requesting the data
Types of analyses: Any purpose
Mechanisms of data availability: With a signed data access agreement
